# Benchmarking and Improving Foundation Model Dietary Estimates from Meal Images

**DOI:** 10.1145/3765612.3767255

**Published:** 2025-12-10

**Authors:** Yongcheng Mu, Jiangwen Sun, Jing He

**Affiliations:** Department of Computer Science, Old Dominion University, Norfolk, VA, United States; Department of Computer Science, Old Dominion University, Norfolk, VA, United States; Department of Computer Science, Old Dominion University, Norfolk, VA, United States

**Keywords:** Image-based Nutrition Estimation, Deep Learning, Large Multimodal Models, Foundation Models

## Abstract

Accurate quantifying dietary contents, such as calories, proteins, carbohydrates, and fats, from an image of a meal plate is vital for managing diabetes. Recently, Large Multimodal Models (LMMs) have excelled in complex vision-language tasks due to their use of very large, highly diverse data. This study benchmarked the use of seven LMMs that include full and lightweight models of GPT, Gemini, and Llama for nutrition estimation based on Google’s Nutrition5k dataset and our own phone-collected DonateAndLearn dataset. We analyzed the performance of LMMs and the RGB-D fusion model, in which the RGB-D model was specifically trained using Nutrition5k data. On our DonateAndLearn dataset, the full-weight versions of LMMs significantly outperformed the RGB-D fusion model, suggesting superior generalization capacity of the LMMs.

We propose a method to integrate Nutrition5k images with phone-collected meal images that often lack the physical sizes of objects in the images. We applied scaled phone images to the RGB-D fusion model to predict the total weight of food in each phone-collected image. Using the predicted weight, the mean absolute percentage error (MAPE) of carbohydrate prediction using the Gemini 2.5 Flash model decreased from 56.6% to 39.5%, based on 78 test cases in the DonateAndLearn dataset. Furthermore, when the ground-truth food weight was provided to Gemini2.5 Flash and GPT-4.1, the MAPE further improved dramatically to 20.2% and 26.8%, respectively, which underscores the critical value of integrating physical information into dietary assessment tools.

## 1 Introduction

Accurate and automated assessment of nutrition intake, such as calories, proteins, fats, and carbohydrates, from a meal image is a vital tool for personal health management, particularly for individuals managing chronic conditions such as diabetes. Although many methods involve estimating food volume, commonly used mobile applications, such as Snap-n-Eat and DepthCalorieCam [1, 2], are limited to specific food categories, each with predefined calorific and nutrition density. Quantifying food nutrition content from an image is challenging due to the three-dimensional nature of food, the variety of food items, and the lack of actual size information in the image. Stereo-based approaches use images to reconstruct the three-dimensional volume of food [3, 4]. However, they often rely on the existence of a fiducial in the image for physical size information. Recent advancements in deep learning and camera sensor technology have demonstrated significant potential in food quantification [5–8]. Models trained on the Nutrition5k dataset [6], in which meal images are paired with depth images (RGB-D), have achieved high accuracy when evaluated on test data captured under controlled conditions similar to the training set [6, 8]. Specifically, the RGB-D fusion network [8] is designed to estimate food nutrition by fusing meal image and depth image feature maps. Although the Nutrition5k dataset is a rich resource of images, its overhead and depth images were captured using a camera fixed on a frame. The resulting images represent those captured at the same distance, from the same camera, and in the same lighting environment. End-to-end models trained on this dataset may not be generalizable to real-world images, including those collected from phones.

The emergence of foundation models presents a potential solution for a generalized model that applies to diverse meal images [9–12]. Having been pre-trained on vast and highly diverse datasets, foundation models have demonstrated exceptional capabilities in a wide range of complex vision-language tasks. Their inherent robustness may allow them to overcome the limitations of specialized models when analyzing less constrained, real-world meal images. Existing research has demonstrated the advantages of foundation models in this domain. NutriBench [13] utilized the collected meal descriptions to evaluate Large Language Models for nutrition estimation. FoodLMM [14] does not use depth information in training because of the challenges in acquiring depth images in real-world scenarios. Reasoning-Driven Food Energy Estimation [15] proposed approaches for fine-tuning and using volume-aware reasoning.

This study aims to benchmark the performance of foundation models for dietary assessment by directly comparing a specialized RGB-D network with several state-of-the-art LMMs. To facilitate this, we conduct experiments on two distinct datasets: the publicly available Nutrition5k dataset, which features controlled lab-quality images, and our own DonateAndLearn dataset, which is composed of images collected from real-world settings via phones.

Our contributions are as follows:

1. We benchmark variants of seven LMMs against an RGB-D fusion model on a popular dataset, Nutrition5k, and a real-world dataset we collected, DonateAndLearn. We demonstrate the superior generalization capability of LMMs, which significantly outperform the specialized RGB-D fusion model on the real-world DonateAndLearn dataset.
2. We provide a method to scale a phone-collected image using its associated depth image. The scaled images are suitable for the RGB-D fusion model food weight prediction.
3. We quantify the effect of providing weight information. Incorporating food weight predicted using the RGB-D fusion model as an input to the LMMs enhances the predictive accuracy for the Gemini2.5 Flash model. To our knowledge, this study is the first to extend the RGB-D fusion model that was originally trained using fixed-camera-collected Nutrition5k data to phone images, which are often collected at varying distances and angles. By leveraging the predicted weight, we show the potential of improving LMMs in real-world, smartphone-collected images.

## 2 Materials and Methods

### 2.1 Nutrition5k Dataset and DonateAndLearn Dataset

**Figure 1.**
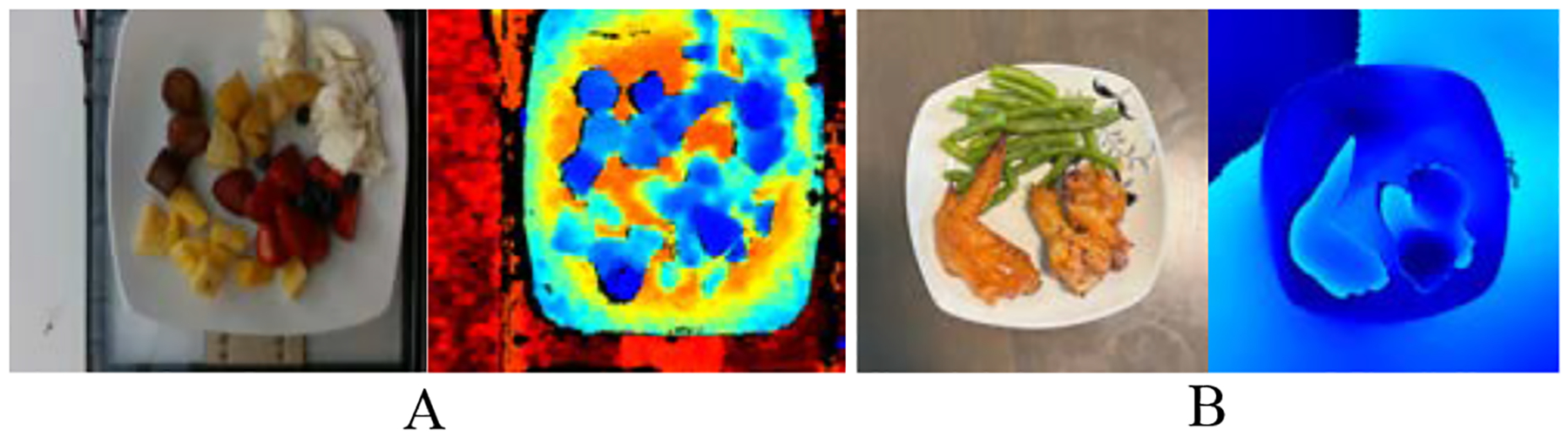
Examples of meal images (left) and the associated depth images (right) of the Nutrition5k dataset (A) and the DonateAndLearn dataset (B).

Two datasets, Nutrition5k and DonateAndLearn, were used in the development and evaluation of the models. Nutrition5k is a large dataset that contains videos and overhead images of meals (Figure 1A) [6]. The overhead images and the corresponding depth images are captured by an Intel RealSense D435 camera fixed at a distance of 35.9 cm from the capture plane [6]. Each dish has a full breakdown of ingredient labels, including calories, mass, fats, carbohydrates, and proteins.

We collected the DonateAndLearn dataset using the DonateAndLearn app we developed for phones (Figure 1B), which is convenient for taking pictures in various places. We collected 78 meal images and their associated depth images using an iPhone 14 Pro and an iPhone 15. Each component of a meal was weighed, and each meal image was labeled with the amount of calories, proteins, fats, and carbohydrates translated from the weight. DonateAndLearn Data contain diverse kinds of foods, such as meats, vegetables, fruits, grains, and nuts. There was a large variation across images in the serving sizes and the quantity of individual nutrients. The mean mass was 137.9 g, with a standard deviation of 80.8 g, and the mean fat was 7.9 g, with a standard deviation of 11.2 g. To assist in taking overhead images, the app contains a levelness indicator and a bounding box for users. The DonateAndLearn app produces the RGB image, depth image, and metadata, including the field of view of the camera, image size, depth range, and user-provided weight of each food component. The DonateAndLearn app is intended to be distributed online for a community effort of data collection.

### 2.2 Development of an RGB-D Fusion Model and Application Using Phone-collected Images

Nutrition5k is a much larger dataset than DonateAndLearn. We used 3,261 pairs of overhead food images and the corresponding depth images in Nutrition5k to develop an RGB-D fusion model using the network in Shao et al. [8]. To explore the generalization of the RGB-D model to phone-collected images, we preprocessed both types of images to reduce the discrepancy between the two. The main component of the preprocessing is the scaling of the phone-collected images (see Section 2.2.2) to match the pixel size of the Nutrition5k image on the reference plane.

#### 2.2.1 Data preprocessing and RGB-D fusion model development.

**Figure 2.**
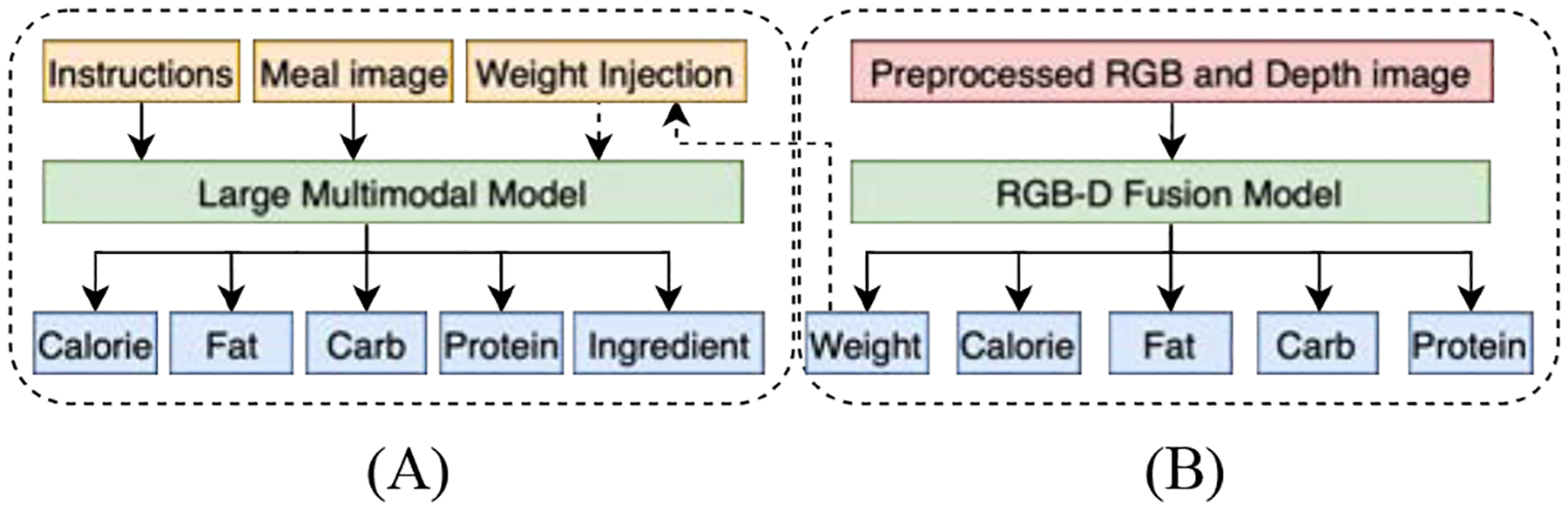
Workflow of the nutrition estimate using the RGB-D fusion model (B) and LMMs (A).

As Nutrition5k overhead images were collected using a fixed-distance camera, the depths in the depth image were limited to a smaller range than those in a DonateAndLearn depth image collected using a mobile phone. We first isolated a bounding box of a meal plate using Grounding DINO [16] to detect the plate. A bounding box was applied to mask the meal image and the raw depth image. We then corrected the artifacts of the missing data in the depth image using the inpainting Telea method in the OpenCV library [17]. Finally, we converted the masked raw depth image into a colored depth image. We then used the network in [8] to train a model on 2,756 pairs of the preprocessed Nutrition5k overhead images and their corresponding depth images. The model was evaluated with 505 pairs of preprocessed Nutrition5k data and 78 pairs of the preprocessed DonateAndLearn dataset. As shown in Figure 2 (B), the RGB-D fusion model takes the preprocessed RGB and depth images as inputs and outputs the amount of weight, calorie, fats, carbohydrates, and proteins.

#### 2.2.2 Scaling of DonateAndLearn images.

In this section, we describe a scaling method to match the pixel size of the reference plane in the Nutrition5k overhead image and the phone-collected image. Initially, a bounding box was detected using the Grounding DINO model, similar to what was done to a Nutrition5k image. Subsequently, we expanded the box by 20 pixels on each side to better capture the surrounding area of a capture plane that often represents a table supporting the meal plate. In the Nutrition5k data collection, the device’s work plane supports a meal plate, and it is considered the capture plane. Similarly, the expanded bounding box was created for the corresponding depth image. The main idea of scaling is to use the camera hardware parameter, Field of View (FOV), and the depth distance of the capture plane to calculate the physical size (in cm) of the image width. Once the physical size of the width is known, the pixel size that represents the surface area per pixel can be calculated. The depth of the capture plane in a DonateAndLearn image was approximated using the average depth within the expanded bounding box, not inside the plate. The rescale factor (3) between a DonateAndLearn image and a Nutrition5k image is defined as follows:

(1) $$\text{PW}=2\times CD\times \text{tan}(FOV_{\text{horizantal}}/2)$$

(2) $$\text{PA}={\left(\frac{\text{PW}}{W}\right)}^{2}$$

(3) $$\text{scale\_factor}=\sqrt{\text{PA/}RA}$$

where *CD* denotes the depth distance between the camera and the capture plane, *FOV*_*horizantal*_ is the horizontal field of view of the camera, *PW* is the physical width of the plane at the capture distance *CD*, *W* is the image width, and *PA* is the physical per-pixel surface area. RA is the reference per-pixel surface area (5.957 × 10^−3^) cm^2^ of the overhead image at the captur distance of 35.9 cm [6]. The meal and depth images were masked, with the depth images colored similarly to Nutrition5k. The images were cropped or padded to match the Nutrition5k resolution of 640 × 480 pixels, based on the computed scale factor. Finally, the preprocessed 78 meal images and depth images were used for evaluation.

### 2.3 Nutrition Estimate Using LMMs

The evaluation involved seven LMMs, including open-source models (Gemma3 27B IT [11], Llama4 Maverick, and Llama4 Scout [10]) and closed-source models (Gemini2.5 Flash [9], GPT-4.1, and GPT-4.1 mini [12]). Gemma3 models are popular, state-of-the-art, lightweight foundation models that run efficiently on a single GPU. Unlike the flagship model GPT-4.1, GPT-4.1 mini is smaller, optimized for efficiency, faster, and more cost-effective. Despite its size, GPT-4.1 mini outperforms the previous flagship, GPT-4o, on various benchmarks. GPT-4.1 nano is a lightweight model distilled from GPT-4.1, while Llama4 Scout is a smaller model than Llama4 Maverick.

To evaluate the LMMs, we used 505 meal images from the Nutrition5k dataset and 78 meal images from the DonateAndLearn dataset. The input is meal images with a maximum dimension of 1024 pixels without preprocessing. The output is a formatted JSON file containing predicted values for calories, fats, carbohydrates, proteins, and ingredients, as shown in Figure 2 (A). We tested the effect of using weight information on LMMs using three settings: no prior knowledge of meal weight, using actual weight, and using weight predicted from the RGB-D fusion model. Detailed LMM instructions are provided in the supplementary document.

## 3 Experimental Results

We evaluated the LMMs and the RGB-D fusion model using the Nutrition5k and DonateAndLearn datasets. The test for LMMs was performed using 505 Nutrition5k images and 78 DonateAndLearn images. The test for the RGB-D model used additional depth images, as the model was trained using both overhead images and depth images. The evaluation included the performance of the models, the effect of using weight in LMMs, and the performance of lightweight LMMs. We further conducted Wilcoxon signed-rank tests and calculated bootstrapping confidence intervals for our model evaluation (see Supplementary 1,2). The percentage of mean absolute error (MAPE) was used to evaluate the accuracy of the predicted caloric and macronutrient content. The MAPE is defined as follows:

(4) $$\text{MAPE}=\frac{\sum_{i=1}^{N}\left|y_{i}-{\hat{y}}_{i}\right|}{\sum_{i=1}^{N}y_{i}}\times 100\%$$

where ***N*** is the number of images, ***y***_***i***_ is the ground-truth value of a nutrient, and ${\hat{y}}_{i}$ is the corresponding predicted value. Calorie values are reported in kilocalories and grams, respectively.

### 3.1 Performance on the Nutrition5k and DonateAndLearn Datasets Across the RGB-D Fusion Model and LMMs (Gemini2.5 Flash, GPT-4.1, and Llama4 Maverick)

**Figure 3.**
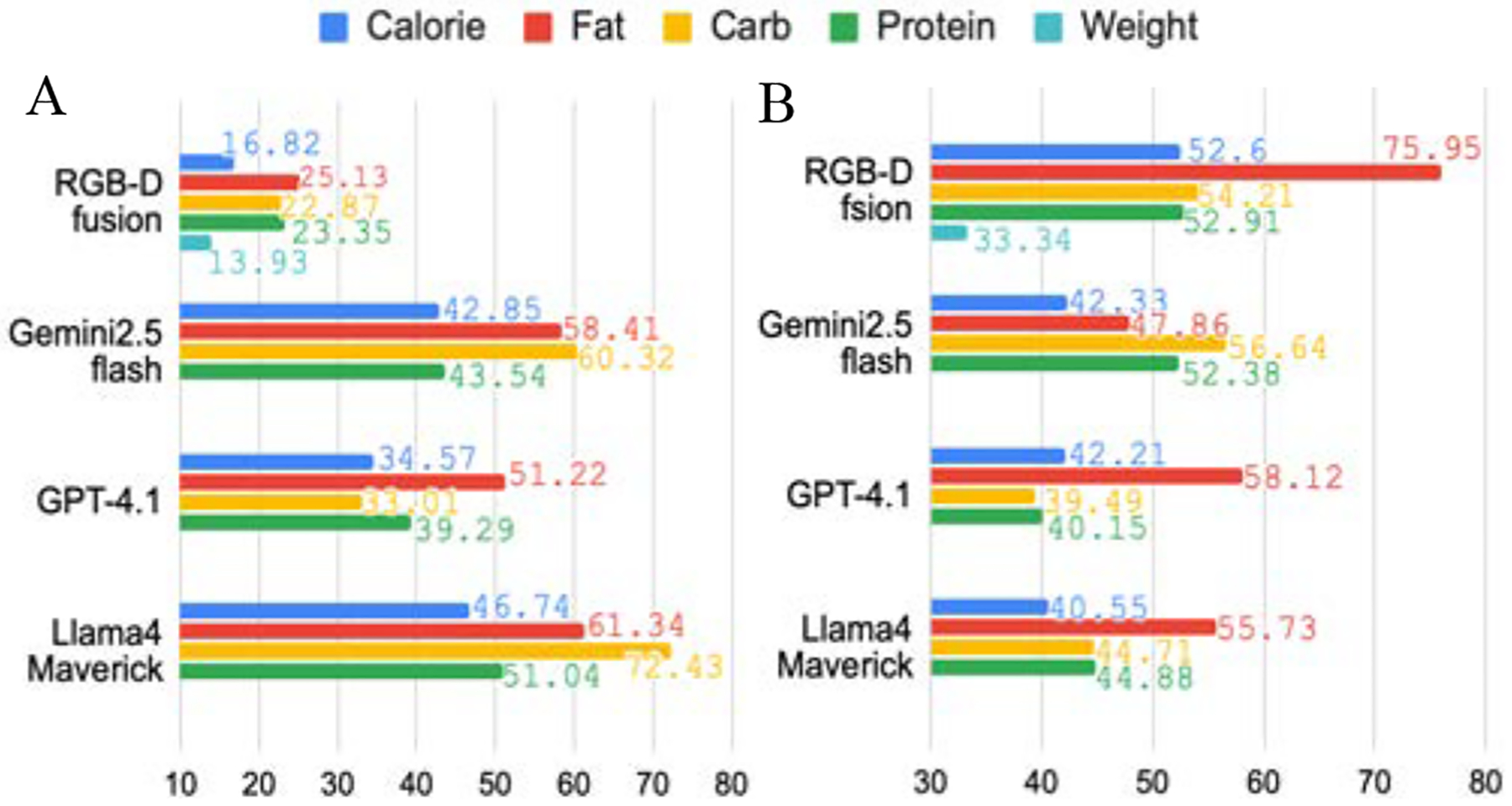
MAPE (%) of the four models: RGB-D fusion, Gemini2.5 Flash, GPT-4.1, and Llama4 Maverick LMMs. The prediction was measured using the Nutrition5k dataset (A) on the DonateAndLearn dataset when no meal weight was used in predicting (B) four nutrients and their weights (shown in cyan).

In this section, we analyze the performance of three “heavy” LMMs and the RGB-D fusion model. In the first experiment, the Nutrition5k dataset was used, while the second experiment was based on the DonateAndLearn dataset. In Experiment 1 (Figure 3(A)), it was not surprising that the RGB-D fusion model performed much better than the LMMs (Gemini2.5 Flash, GPT-4.1, and Llama4 Maverick) in all four nutrition categories. The advantage of the RGB-D fusion was likely the specific use of Nutrition5k data in the training of this model. For example, the MAPE of calories for RGB-D fusion was only 16.82, but the best of the three LMMs (GPT) was 34.57% (Figure 3(A)). This suggests that there is still room for LMMs to improve. GPT-4.1 demonstrated relatively superior performance among the three LMMs in all four nutrition categories, notably the prediction of carbohydrates, achieving 33.01%, while Gemini2.5 Flash and Llama4 Maverick achieved only 60.32% and 72.43%, respectively. In Experiment 2, in which the DonateAndLearn dataset was used, a starkly different picture was observed. All three LMMs exhibited significantly superior MAPE compared with the RGB-D fusion model (Figure 3(B)) across all four categories, with the exception of carbohydrate prediction in Gemini2.5 Flash. To further evaluate the statistical significance of the results, the Wilcoxon tests revealed (Supplementary Table S1) that LMMs statistically outperformed the RGB-D fusion model on the DonateAndLearn data, except for Gemini2.5 Flash and Llama4 Maverick on carbohydrate and protein contents. Combining the evaluation using MAPE, the Wilcoxon test, and bootstrapping, GPT-4.1 outperformed the RGB-D model in all four nutrient categories. Gemini2.5 Flash and Llama4 Maverick outperformed the RGB-D model in calorie and fat prediction.

For example, all three LMMs had MAPE of calorie between 40.55% and 42.33%, while the RGB-D model had a much higher value of 52.6% (Figure 3(B)). It appears that all three LMMs had a consistent range of MAPE in calorie prediction in both the Nutrition5k data (34.57%–46.74%) and the DonateAndLearn data (40.55%–42.33%) (Figure 3 A, B). However, the RGB-D model, which was trained using Nutrition5k data, did not seem generalizable to the DonateAndLearn data. Its calorie prediction ranged from 16.82% to 52.6% MAPE between the two datasets.

### 3.2 The Effect of Using Weight on LMMs for Evaluating Carbohydrates

**Figure 4.**
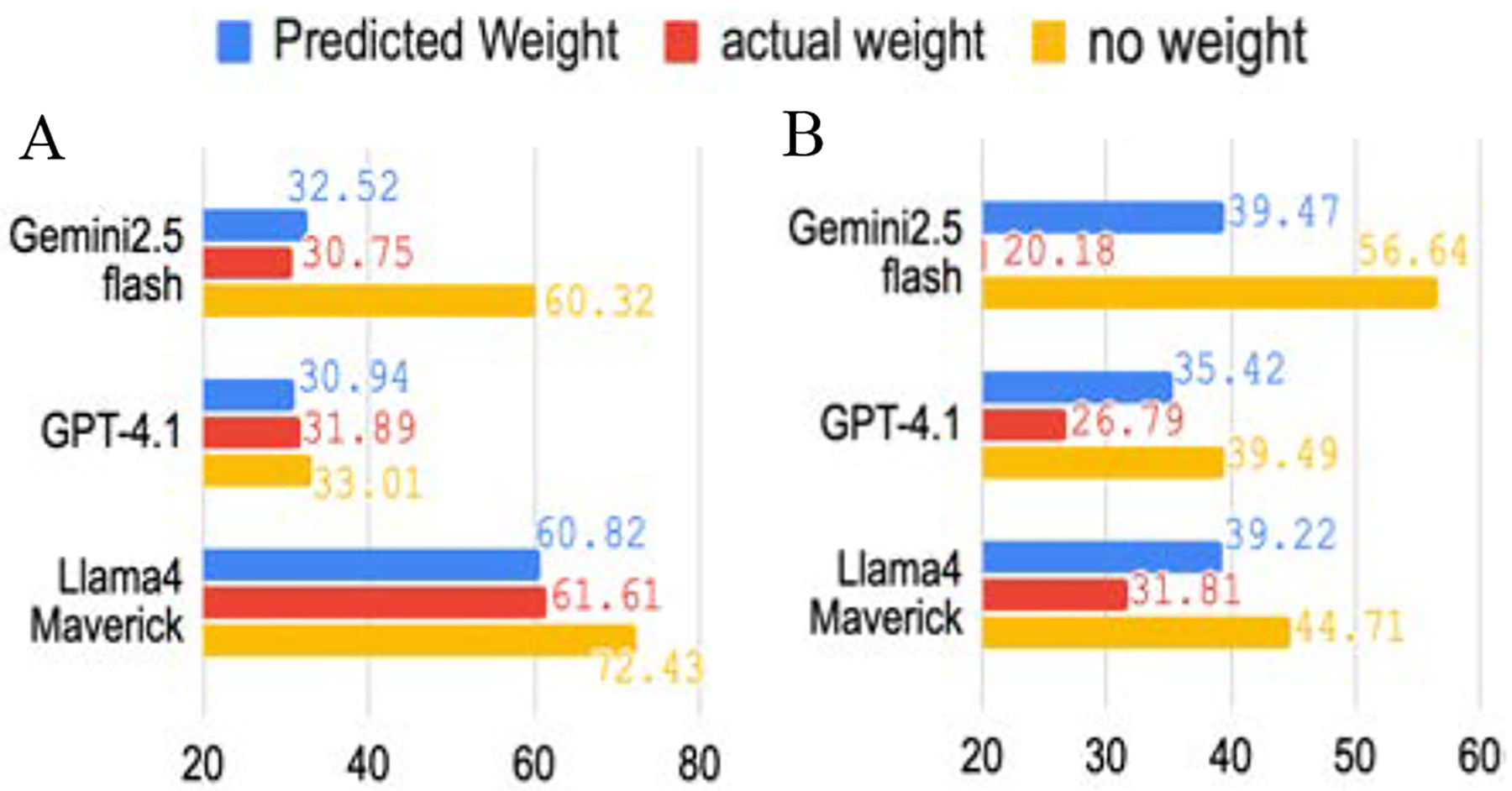
Effect of using weight on carbohydrate estimation for the three models: Gemini2.5 Flash, GPT-4.1, and Llama4 Maverick. (A) Performance on the Nutrition5k dataset; (B) Performance on the DonateAndLearn dataset. The MAPE (%) without using the weight information in prediction (yellow), using the actual weight of the meal (red), and using the predicted weight using RGB-D fusion (blue).

As carbohydrate content is related to the weight of a meal, we analyzed the effect of using weight information in assisting three heavy LMMs. As expected, using the correct weight information made the prediction more accurate than using no weight information for all three LMMs in the Nutrition5k (Figure 4 (A)) and DonateAndLearn datasets (Figure 4 (B)). Although the MAPE decreased significantly for Gemini2.5 Flash and Llama4 Maverick in the Nutrition5k data, the level of improvement for GPT-4.1 was minimal, suggesting that GPT-4.1 already predicted carbohydrates effectively without the need for weight information for the Nutrition5k data. When evaluated using the Nutrition5k data, using the predicted weight reduced the MAPE for all three LMMs, similar to the effect of using the actual weight information (Figure 4 (A)). The use of actual weight and predicted weight yielded remarkably similar performance, which was attributed to the highly accurate weight prediction (13.93% in Figure 3 (A)) in the RGB-D fusion model. Gemini2.5 Flash seemed to behave differently from Llama4 Maverick, as using either the actual weight or the predicted weight drastically reduced the MAPE from 60.32% to 30.75% or 32.52%, but it only reduced the MAPE from 72.43% to 60.82% or 61.61% of Llama4 Maverick (Figure 4 (A)). Nevertheless, there was still a significant amount of error in carbohydrate prediction.

Although we observed similar effects of using weight on the DonateAndLearn dataset (Figure 4(B)), the effect appeared to be more than that observed for Nutrition5k when the actual weight was used (red vs. yellow in Figure 4(B)). This suggests the challenges in phone-collected meal images, in which different images have different scales. By providing the actual weight, all three LMMs significantly improved the prediction accuracy, particularly for Gemini2.5 Flash, with the MAPE decreasing from 56.64% to 20.18%. As shown in Table S2, Gemini2.5 Flash demonstrated statistically significant outperformance in predicted weight compared with no weight. Conversely, GPT4.1 and Llama4 Maverick did not exhibit a statistically significant difference between no weight and predicted weight.

Regardless of the various effects of the three LMMs, the weight information provided to LMMs was found to be important for carbohydrate prediction in our studies. Specifically, the results showed different levels of effect when the predicted weight was at a different accuracy. It appears that the more accurate the weight used, the greater the overall enhancement. When the predicted weight was reduced to a MAPE of 33.93%, its effect decreased, as shown in the results for the DonateAndLearn data. Weight represents a physical metric of a meal and is important in the LMM prediction of carbohydrates. It would be interesting to see if other physical metrics of a meal could enhance the estimates of other nutrients. In our study, we observed that weight information had little effect on estimating calories and fats, but it affected most in the estimate of carbohydrates and next to the proteins (Table 2).

### 3.3 Performance of Lightweight Models

We conducted experiments involving seven LMMs on the Nutrition5k dataset (Table 2). We used three lightweight models, three heavyweight models, and an optimized model, GPT-4.1 mini (distilled from GPT-4.1), without providing physical weight information. Based on the MAPE values (Table 2) and the Wilcoxon test (Supplementary 1.2.3), more advanced (heavyweight) LMMs exhibited a distinct advantage over lightweight models.

Compared with the smaller, well-optimized GPT-4.1 mini, GPT-4.1 demonstrated slightly better performance in estimating fats (51.2%), carbohydrates (33.0%), and proteins (39.3%). Conversely, GPT-4.1 mini exhibited a slight advantage in calorie estimation, achieving a MAPE of 32.7% compared with 34.6% for GPT-4.1 on the Nutrition5k dataset. The results indicate a clear advantage of more advanced, heavyweight models over lightweight models without providing physical weight information. However, a well-optimized model can be more efficient while maintaining its performance, such as GPT-4.1 mini.

## 4 Discussion

Our study highlights a significant leap forward in automated nutritional analysis by demonstrating the superior generalization capabilities of LMMs over the RGB-D fusion model for real-world applications. Although the RGB-D fusion model has shown promise, it is limited to images similar to the training data. This study addresses this challenge by showing that LMMs trained on extensive and diverse internet-scale image datasets with varying qualities, compositions, and contexts can effectively manage variability captured by standard mobile devices, such as those in the DonateAndLearn dataset. The lack of generalizability of the RGB-D fusion model is likely due to variations in depth data between the datasets. Specifically, an Intel RealSense D435 sensor used in the Nutrition5k dataset can capture a depth resolution up to 1280 × 720. Most mobile devices capture depth maps at a much lower resolution (768 × 576). Lighting conditions also affect the quality of depth maps, especially in lower-quality camera sensors. The angle of capturing may also influence depth maps. For instance, Figure 1 (B) shows a darker blue region in the top-left corner due to the tilt of the capturing device, while depth maps in the Nutrition5k dataset are all captured under the same conditions using the same sensor.

Another critical finding is the enhancement of accuracy when physical information is integrated with visual data. By providing the predicted total food weight to the LMMs, the carbohydrate estimation significantly improved; for example, Gemini2.5 Flash achieved 39.47%, while only 56.64% was achieved on the DonateAndLearn dataset with no weight. This emphasizes a key limitation of relying solely on visual data for nutritional estimation; without a sense of size, depth, or weight, even advanced models struggle to provide precise dietary assessments. This result strongly advocates a hybrid approach in future dietary assessment tools, one that combines the powerful image interpretation of LMMs with reliable physical information derived from depth images.

Among the compared models, the RGB-D fusion model had the smallest size, with about 110 million trainable parameters. All LMMs were larger. Although the size of Gemini 2.5 Flash and GPT models has not been made public, the models are projected to have at least tens or hundreds of billions of parameters. Gemma3 is likely the lightest LMM in our study, with about 27 billion parameters. Llama 4 Maverick and Scout are much larger, with 400 billion and 109 billion, respectively. Larger models take longer to make predictions. In our current workflow, LMMs use APIs from companies such as OpenAI API [18], Google AI Studio [19], and Llama API [20] (see Supplementary 1.1); thus, execution occurs on their servers. In the future, we may consider techniques such as distilling to create smaller models that run locally on mobile devices.

These findings have significant implications for personal and public health. For individuals managing chronic conditions such as diabetes, an accurate and user-friendly tool that estimates nutritional content from a smartphone photo could improve dietary adherence and glycemic control. Beyond individual health, such a tool could be used for public health research to collect population-level dietary data. This capability could yield novel insights into nutritional trends, the efficacy of dietary interventions, and the correlations between diet and chronic diseases across diverse populations.

## 5 Conclusions

This study demonstrates the superior ability of LMMs to generalize the nutritional analysis of food images in real-world settings compared with the RGB-D fusion model trained on the Nutrition5k dataset. LMMs significantly outperformed the trained RGB-D fusion model on a newly collected dataset of real-world food images, DonateAndLearn.

This study further highlights that the accuracy of LMMs in carbohydrate estimation can be dramatically improved by incorporating physical data such as the total weight of food. When the predicted weight of a different accuracy was provided alongside the image, the accuracy of LMMs in carbohydrate estimation was affected at different levels. Ultimately, this study underscores two critical points for the future of automated dietary assessment. First, the properly selected LMM models provide a distinct advantage in handling the variability of real-world meal images. Second, to build highly accurate dietary assessment tools, the integration of physical information, such as food weight, is valuable.

## Supplementary Material

SI

## Figures and Tables

**Table 1.** Effect of using weight on the estimates of calories, fats, carbohydrates, and proteins for the three foundation models: Gemini2.5 Flash, GPT-4.1, and Llama4 Maverick. The MAPEs of a foundation model in different settings are shown in each cell, from left to right, with no prior weight knowledge, with the use of predicted weight, and with the use of the actual weight of the food. The first three rows are evaluated on the Nutrition5k dataset, while the subsequent three rows, highlighted in gray, represent the evaluation on the DonateAndLearn dataset.

Model	Calories	Fats	Carbohydrates	Proteins	Mean
Gemini2.5 Flash	42.9/29.6/29.5	58.4/53.3/55.1	**60.3/32.5/30.8**	**43.5/33.0/32.3**	51.3/37.1/36.9
GPT-4.1	34.6/28.4/25.1	51.2/50.6/47.7	**33.0/30.9/31.9**	**39.3/30.4/27.2**	39.5/35.1/33.0
Llama4 Maverick	46.7/39.8/37.8	61.3/62.4/60.3	**72.4/60.8/61.6**	**51.0/43.4/43.6**	57.9/51.6/50.8
Gemini2.5 Flash	42.3/39.8/17.3	47.9/50.8/38.2	**56.6/39.5/20.2**	**52.4/35.2/28.2**	49.8/41.3/26.0
GPT-4.1	42.2/44.4/25.5	58.1/61.0/44.8	**39.5/35.4/26.8**	**40.2/35.4/22.1**	45.0/44.1/29.8
Llama4 Maverick	40.6/46.5/23.8	55.7/63.4/40.0	**44.7/39.2/31.8**	**44.9/42.7/32.4**	46.5/48.0/32.0

**Table 2.** The MAPE (%) of nutrient estimation for seven foundation models using the Nutrition5k dataset: three lightweight models (Gemma3 27B IT, GPT-4.1 nano, and Llama4 Scout), three heavyweight models (highlighted in gray), and an optimized model (GPT-4.1 mini).

Method	Calories	Fats	Carbohydrates	Proteins	Mean
Gemma3 27B IT	54.5	61.7	87.1	55.2	64.6
Gemini2.5 Flash	42.9	58.4	60.3	43.5	51.3
GPT-4.1 nano	43.9	56.8	83.9	54.5	59.8
GPT-4.1 mini	32.7	52.0	36.7	41.5	40.7
GPT-4.1	34.6	51.2	33.0	39.3	39.5
Llama4 Scout	72.7	85.5	91.0	76.7	81.5
Llama4 Maverick	46.7	61.3	72.4	51.0	57.9

## CCS CONCEPTS

• AI and Machine Learning in Healthcare • Large Language Model (LLM) for Health
