# Supplementary material for: Benchmarking and Improving Foundation Model Dietary Estimates from Meal Images
Source: ACM BCB. Author manuscript; Available in PMC 2026 Jul 26. (PMC13401436; doi:10.1145/3765612.3767255)
Supplement: SI [file NIHMS2196004-supplement-SI.pdf]

Supplementary Material

Benchmarking and Improving Foundation Model Dietary Estimates from Meal Images

Yongcheng Mu, Jiangwen Sun, and Jing He†

Department of Computer Science, Old Dominion University— Norfolk, VA 23529, USA

†Correspondent Author: Jing He, jhe@cs.odu.edu.

1.1 The use of LMM APIs

For the evaluation of the LMMs, we used several APIs. OpenAI API [1] was employed for the evaluation of GPT models. Google AI Studio [2] was used for Gemini 2.5 Flash and Gemma 3. The Llama API [3] was used for Llama 4 evaluations. Figure S1 (A) illustrates the input of the GPT models. Figure S1 (B) serves as a guiding text prompt for the prediction of LMMs. It incorporates weight information (as per Instruction 2) and formats the predictions in JSON structure.

```
messages = [
    {
        "role": "user",
        "content": [
            {
                "type": "text",
                "text": prompt
            },
            {
                "type": "image_url",
                "image_url": {
                    "url": f"data:image/jpeg;base64,{image_data}"
                }
            }
        ]
    }
]
```

(A)

```
"""
INSTRUCTIONS:
1. Identify all visible food items in the image
2. "IMPORTANT: The total weight of the food items only (excluding
plate, utensils, and other non-food items) is {weight_info:.1f} grams.
Use this EXACT weight for your calculations. Do not estimate weight -
use the provided {weight_info:.1f}g."
3. Calculate total nutritional values for ALL food shown
4. Provide EXACT NUMBERS only (no ranges, no "approximately")
5. Use standard nutritional database values (USDA FoodData Central)
6. COMPLETE the entire JSON structure - do not truncate

Return complete JSON structure:

{
  "food_items": ["detailed list of identified foods"],
  "calories": exact_number,
  "protein_g": exact_number,
  "carbohydrates_g": exact_number,
  "fat_g": exact_number,
  "weight_g": exact_number,
  "analysis_notes": "brief description of food identification and
portion estimation"
}

CRITICAL: Complete the entire JSON structure. All nutritional values in
grams (except calories in kcal). Provide COMPLETE response - do not
stop mid-sentence.
"""
```

(B)

Figure S1. An example of the input message to GPT models (A), and text prompt used in the message (B).

1.2 Wilcoxon tests and Bootstrapping confidence intervals for model evaluation

We conducted Wilcoxon signed-rank tests using functions in the scipy.stats module (SciPy v1.13.1) [4]. To determine the confidence intervals, we used the bootstrap technique. The approach performs 2,000 bootstrap resampling iterations with replacement from the original data, calculating the median for each sample. The confidence interval bounds were subsequently determined using the percentile method, specifically the 2.5th and 97.5th percentiles for 95% confidence intervals.

1.2.1 *Wilcoxon tests for the comparisons between the RGB-D fusion model and each of the three LMMs.* Table S1 contains statistical test results related to those in Figure 3 in the paper. The outperformance of the RGB-D fusion model against the three LMMs on the Nutrition5k dataset showed statistical significance, with p-values all well below 0.05 and negative confidence intervals (Table S1). Its underperformance on the DonateAndLearn dataset showed statistical significance in the estimates of calories and fats for all three LMMs and on all four nutrition categories for GPT-4.1. The RGB-D model does not show statistical significance, using the DonateAndLearn dataset, against Gemini2.5 Flash and Llama 4 Maverick for predicting carbohydrate and protein (with a p-value above 0.05, Table S1). When all four nutrition categories are considered, the mean p-values are all below 0.05, suggesting the RGB-D model underperforms each of the three LMMs (last column of Table S1).

Table S1. Wilcoxon test p-values and the bootstrapping confidence intervals (CI) for comparing the RGB-D model and each of the three LMMs. The first three rows are results obtained using the Nutrition5k dataset, and the last three rows, highlighted in gray, are those using the DonateAndLearn dataset. P-value (upper) and CI (lower) are shown in each cell.

Comparison	P-value and Bootstrapping Confidence Interval [CI_Lower, CI_Upper]				
	Cal	Fat	Carb	Protein	Mean
RGB-D Fusion vs. Gemini2.5 Flash	~ 0.000000 [-35.7261, -19.2257]	~ 0.000000 [-2.0730, -0.4398]	~ 0.000000 [-3.5375, -2.1862]	~ 0.000000 [-1.2902, -0.6181]	~ 0.000000 [-11.8950, -6.2651]
RGB-D Fusion vs. GPT-4.1	~ 0.000000 [-32.7242, -19.3443]	~ 0.000000 [-1.8544, -0.8273]	~ 0.000000 [-1.5292, -0.6055]	~ 0.000000 [-0.8881, -0.3120]	~ 0.000000 [-9.8955, -6.4411]
RGB-D Fusion vs. Llama4 Maverick	~ 0.000000 [-55.5354, -41.1725]	~ 0.000000 [-2.5294, -0.8892]	~ 0.000000 [-7.8036, -5.4576]	~ 0.000000 [-2.8970, -1.8323]	~ 0.000000 [-17.5756, -12.7490]
RGB-D Fusion vs. Gemini2.5 Flash	0.023589 [0.6809, 16.8779]	0.000028 [0.2485, 1.5131]	0.363357 [-1.0363, 2.3263]	0.257163 [-0.1713, 1.0878]	0.016700 [0.0562, 5.4652]
RGB-D Fusion vs. GPT-4.1	0.000616 [5.2807, 27.1773]	0.000112 [0.2441, 1.3561]	0.001472 [0.4267, 2.9176]	0.002920 [0.4122, 1.5541]	0.000244 [2.2972, 8.2810]
RGB-D Fusion vs. Llama4 Maverick	0.026833 [1.7798, 22.2115]	0.000193 [0.2526, 1.6200]	0.215821 [-1.4810, 3.0585]	0.208522 [-0.2374, 1.1618]	0.012148 [1.2243, 7.3652]

1.2.2 *Wilcoxon tests for the effect of using weight in LMMs for carbohydrate quantification*. This section and Table S2 contain statistical analysis for the results in Figure 4(B) of the paper. The Wilcoxon test (NoWeight vs. PredictedWeight) was performed to compare the results obtained by each LMM between two settings. In the first setting, no weight was used in the LMM prompt. In the second setting, the predicted total weight of food, which was obtained from the RGB-D model, was used in the prompt of an LMM. Similarly, the Wilcoxon tests were also conducted in the settings of (NoWeight vs. ActualWeight) and (ActualWeight vs. PredictedWeight). For the Nutrition5k dataset (top rows in Table S2), the use of either actual weight or predicted weight of food improved the accuracy of carbohydrate prediction for all three LMMs, with statistical significance, since all p-values are below 0.05 (Table S2). For the DonateAndLearn dataset, the use of actual weight also showed statistical significance in improving the accuracy of the three LMMs. When the predicted food weight was used in the prompt of the LMMs, Gemini2.5 Flash showed statistical significance in improving the accuracy of carbohydrate estimation, but GPT4.1 and Llama4 Maverick did not show statistical significance.

Table S2. Wilcoxon test p-values and the bootstrapping confidence intervals (CI) for the effect of using weight in LMMs. The top half of the table shows the results when LMMs were applied to the Nutrition5k dataset, while the bottom half (highlighted with gray) shows the results when LMMs were applied to the DonateAndLearn dataset. P-value and CI are shown in each cell.

Model	Comparison	P-value [CI_Lower, CI_Upper]
Gemini2.5 Flash	NoWeight vs. PredictedWeight	~0.000000 [9.9200, 18.9000]
	NoWeight vs. ActualWeight	~0.000000 [1.7000, 2.9420]
	ActualWeight vs. PredictedWeight	0.000113 [-0.5200, -0.1500]
GPT-4.1	NoWeight vs. PredictedWeight	0.003459 [0.0000, 0.7340]
	NoWeight vs. ActualWeight	0.001842 [0.2000, 0.8000]
	ActualWeight vs. PredictedWeight	0.490466 [-0.3000, 0.0000]
Llama4 Maverick	NoWeight vs. PredictedWeight	~0.000000 [1.1500, 3.0600]
	NoWeight vs. ActualWeight	~0.000000 [1.7900, 3.4300]
	ActualWeight vs. PredictedWeight	0.038463 [-0.2890, -0.0200]
Gemini2.5 Flash	NoWeight vs. PredictedWeight	0.015340 [0.0000, 2.3300]
	NoWeight vs. ActualWeight	~0.000000 [1.4400, 3.8301]
	ActualWeight vs. PredictedWeight	~0.000000 [-2.3100, -0.5500]
GPT-4.1	NoWeight vs. PredictedWeight	0.895146 [-0.4000, 0.2000]
	NoWeight vs. ActualWeight	0.000099 [0.2200, 1.8000]
	ActualWeight vs. PredictedWeight	0.000016 [-1.5300, -0.3950]
Llama4 Maverick	NoWeight vs. PredictedWeight	0.252297 [-0.1878, 0.9335]
	NoWeight vs. ActualWeight	0.000069 [0.4800, 2.4440]
	ActualWeight vs. PredictedWeight	0.2937 [-1.1601, -0.1050]

1.2.3 *Wilcoxon tests for the comparisons between lightweight and heavyweight models.* This section and Table S3 provide more statistical analysis, regarding Table 2 of the paper, on the performance between a lightweight and a heavyweight LMM model. The Wilcoxon test results showed that all the heavyweight LMM models outperform lightweight models with statistical significance on Nutrition5k dataset, with the exception of the comparison between

GPT-4.1 and GPT-4.1 nano. However, the outperformance observed for the Nutrition5k dataset did not show statistical significance when the DonateAndLearn dataset was used.

Table S3. Wilcoxon test p-values and confidence intervals (CI) for the comparison between a lightweight model and a heavyweight model. The top half of the table shows the results when LMMs were applied to the Nutrition5k dataset, while the bottom half (highlighted with gray) shows the results when LMMs were applied to the DonateAndLearn dataset. P-value (upper) and CI (lower) are shown in each cell.

Comparison	P-value and Bootstrapping Confidence Interval ([CI_Lower, CI_Upper])				
	Cal	Fat	Carb	Protein	Mean
Gemma3 27B IT vs.<br>Gemini2.5 Flash	~0.000000<br>[13.0000, 28.3380]	0.022321<br>[0.0200, 0.2000]	~0.000000<br>[2.7000, 4.7000]	~0.000000<br>[0.6580, 1.4800]	~0.000000<br>[4.1120, 7.7750]
GPT-4.1 vs.<br>GPT-4.1 mini	0.117315<br>[-2.2890, 11.1015]	0.751101<br>[-0.0560, 0.0000]	0.053219<br>[-0.5120, -0.1000]	0.076986<br>[-0.2720, 0.0000]	0.258728<br>[-0.1640, 1.1750]
GPT-4.1 vs.<br>GPT-4.1 nano	0.000028<br>[-17.5000, -1.0000]	0.193790<br>[-0.0680, 0.0000]	~ 0.000000<br>[-6.0000, -3.0000]	~ 0.000000<br>[-1.0000, -0.4000]	~ 0.000000<br>[-6.7965, -1.2750]
GPT-4.1 mini vs.<br>GPT-4.1 nano	~ 0.000000<br>[-20, -5]	0.710361<br>[0, 0.082]	~ 0.000000<br>[-5, -2.5]	~ 0.000000<br>[-0.8, 0]	~ 0.000000<br>[-7.493, -2.847]
Llama4 Maverick vs.<br>Llama4 Scout	~ 0.000000<br>[-11.3860, 0.0000]	0.011399<br>[-0.2042, -0.0100]	0.023057<br>[-0.6900, 0.0600]	~ 0.000000<br>[-1.4200, -0.3900]	~ 0.000000<br>[-3.8250, -1.3210]
Gemma3 27B IT vs.<br>Gemini2.5 Flash	0.718773<br>[-10.0000, 7.9070]	0.252811<br>[0.0000, 0.1360]	0.873330<br>[-1.0027, 1.0902]	0.233814<br>[-0.1500, 0.5000]	0.723491<br>[-3.3000, 1.8130]
GPT-4.1 vs.<br>GPT-4.1 mini	0.452964<br>[-1.0000, 1.4800]	0.661178<br>[-0.2000, 0.0000]	0.938518<br>[-0.1000, 0.1000]	0.114693<br>[-0.4007, 0.0000]	0.566143<br>[-1.0291, 7.4736]
GPT-4.1 vs.<br>GPT-4.1 nano	0.261702<br>[-9.8080, 0.5000]	0.773851<br>[-0.0150, 0.0000]	0.085702<br>[-1.0000, 0.0000]	0.624566<br>[-0.3000, 0.1820]	0.231796<br>[-2.7750, 0.2410]
GPT-4.1 mini vs.<br>GP- 4.1 nano	0.067753<br>[-15, 0]	0.646078<br>[0, 0.2]	0.021784<br>[-1.15, 0]	0.944014<br>[0, 0.223]	0.064956<br>[-2.75, 0.022]
Llama4 Maverick vs.<br>Llama4 Scout	0.808936<br>[-8.0000, 0.0000]	0.664198<br>[-0.1480, 0.0800]	0.093916<br>[-1.6280, 0.0000]	0.097872<br>[-0.3450, 0.0300]	0.677488<br>[-2.6925, 1.2275]

Table S4. The performance of seven foundation models in nutrient estimation. The MAPE (%) of Lightweight models (Gemma3 27B IT, GPT-4.1 nano, and Llama4 Scout) and heavyweight models (Gemini2.5 Flash, GPT-4.1 mini, GPT-4.1, and Llama4 Maverick), on the Nutrition5k dataset (top rows), and DonateAndLearn lower (lower shaded rows) are shown.

Method	Calorie	Fat	Carb	Protein	Mean
Gemma3 27B IT	54.5	61.7	87.1	55.2	64.6
Gemini2.5 Flash	42.9	58.4	60.3	43.5	51.3
GPT-4.1 nano	43.9	56.8	83.9	54.5	59.8
GPT-4.1 mini	32.7	52.0	36.7	41.5	40.7
GPT-4.1	34.6	51.2	33.0	39.3	39.5
Llama4 Scout	72.7	85.5	91.0	76.7	81.5
Llama4 Maverick	46.7	61.3	72.4	51.0	57.9
Gemma3 27B IT	47.2	70.8	56.4	58.2	58.1
Gemini2.5 Flash	42.3	47.9	56.6	52.4	49.8
GPT-4.1 nano	49.4	63.5	55.5	54.8	55.8
GPT-4.1 mini	35.9	53.5	36.0	44.5	42.5
GPT-4.1	42.2	58.1	39.5	40.2	45.0
Llama4 Scout	45.7	57.6	61.9	62.2	56.9
Llama4 Maverick	40.6	55.7	44.7	44.9	46.5

References

[1] OpenAI. 2025. OpenAI platform. Retrieved from https://openai.com/api/.
[2] Google. 2025. Google AI Studio. Retrieved from https://aistudio.google.com/welcome.
[3] Llama. 2025. Llama API. Retrieved from https://llama.developer.meta.com/?team_id=1212050417367470.
[4] Pauli Virtanen, Ralf Gommers, Travis E Oliphant, Matt Haberland, Tyler Reddy, David Cournapeau, Evgeni Burovski, Pearu Peterson, Warren Weckesser, and Jonathan Bright. 2020. SciPy 1.0: fundamental algorithms for scientific computing in Python. *Nature methods* 17, 3, https://doi.org/ https://doi.org/10.1038/s41592-019-0686-2.
